# Biomass to bioenergy transition through sustainable bioconversion and circular bioeconomy strategies

**DOI:** 10.1186/s40643-026-01141-1

**Published:** 2026-11-05

**Authors:** Btissam Niya, Ahmad Mahmud, Abdesamad Ou-douaou, Aziz Boutafda, Rachid Benhida, Mohammed Danouche

**Affiliations:** 1 College of Chemical Sciences and Engineering (CCSE), Department of Chemical and Biochemical Sciences - Green Process Engineering (CBS-GPE), Mohammed VI Polytechnic University (UM6P), 43150, Ben Guerir, Morocco; 2 Institute of Chemistry, Nice UMR7272, Cote d’Azur University, French National Center for Scientific Research (CNRS), Nice, France

**Keywords:** Biomass, Bioenergy, Fermentation, Anaerobic digestion, Life cycle assessments

## Abstract

Access to modern electricity and clean cooking technologies remains uneven across much of Africa, despite the continent’s abundant biomass resources, which have the potential to enhance energy security while supporting the transition toward a circular bioeconomy. However, a substantial proportion of agricultural, forestry, municipal, and marine organic residues is still treated as waste rather than valorized within the formal energy sector. Morocco exemplifies this disconnect. Although the country has achieved near-universal rural electrification, biomass derived from agricultural and marine resources contributes less than 2% of the modern energy mix, highlighting that the principal challenge lies not in resource availability but in the development of efficient conversion pathways and integrated deployment strategies. This review examines recent advances in biomass-to-bioenergy technologies, with particular emphasis on sustainable bioconversion processes, including enzymatic hydrolysis, microbial fermentation, anaerobic digestion, and microbial fuel cells. Beyond evaluating technological progress, we assess research trends, innovation dynamics, and technology transfer through bibliometric and patent analyses to identify leading research areas, emerging technologies, and geographical disparities across Africa. We further discuss the role of life cycle assessment in evaluating the environmental sustainability of biomass conversion pathways and demonstrate its importance in identifying low-carbon alternatives capable of reducing greenhouse gas emissions. We conclude that Africa’s energy sovereignty depends on moving away from generic policies toward integrated, country-specific deployment strategies that prioritize locally available biomass within circular bioeconomy frameworks.

**Graphical abstract:**

**Figure:**
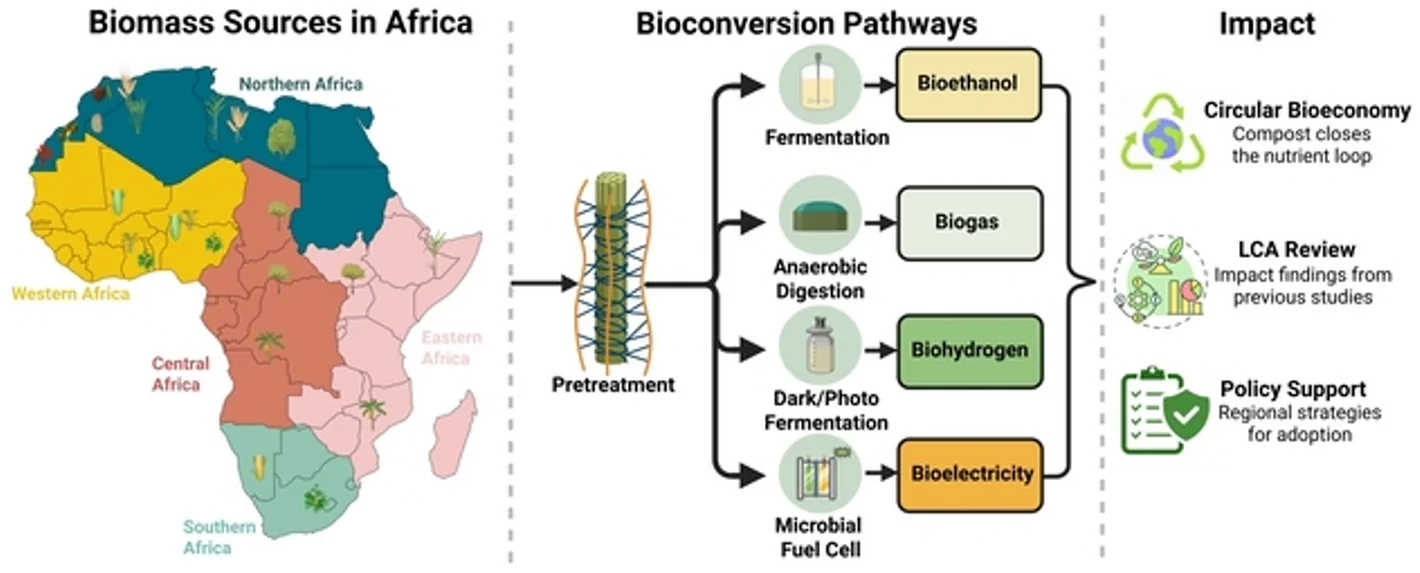

## Introduction

The global shift toward an energy transition is no longer just a climate necessity; it is a fundamental requirement for social equity and clean energy access. In 2023, about 2.1 billion people worldwide still lacked access to clean cooking, and about 565 million people in sub-Saharan Africa remained without electricity (IEA 2023). This situation is driving the adoption of clean energy technologies, with bioenergy emerging as a cornerstone for sustainable development by providing heat, electricity, and fuels from biomass (IRENA 2024). Biomass comprising agricultural residues, forest by-products, energy crops, algal biomass, and organic waste is Africa's most abundant yet underutilized renewable resource. Africa generates several hundred million tonsne of agricultural residues annually, with the largest contributions originating from major agricultural producers such as Nigeria, Ethiopia, Kenya, and Morocco. Yet, this substantial biomass potential remains largely underutilized. In sub-Saharan Africa, 75% of the population continues to depend on traditional biomass for cooking and heating. Such practices are characterized by low energy conversion efficiency and are associated with indoor air pollution, deforestation, and long-term health risks (Mensah et al. 2024; Mensah and Yankson 2025). Morocco provides an illustrative case of this discrepancy. The country generates substantial biomass yields from olive, citrus and date cultivation, yet only 2% is currently integrated into the modern bioenergy mix (Labbi 2017). This suggests that the primary limitation is not resource scarcity, but rather the absence of effective conversion pathways, infrastructure, and enabling policy frameworks. From a broader perspective, biomass valorization not only contributes to energy generation, but also aligns closely with circular bioeconomy principles by enhancing resource efficiency, creating jobs, and supporting rural development (Waqas et al. 2023; Balasubramani et al. 2023). The transformation of biomass through both biochemical and thermochemical conversion pathways contributes to reducing greenhouse gas (GHG) emissions (Kazmi et al. 2025; Rajpoot et al. 2025). Integrated biorefineries are increasingly regarded as cornerstones of the bioeconomy, contributing not only to energy and high-value bio-based products but also to climate change mitigation (Bajpai 2021; Manikandan et al. 2023). Biofuels are categorized into four generations based on feedstock and production methods. First-generation biofuels come from food crops but compete with food supply. Second-generation biofuels offer better sustainability as they are generated from non-food biomass and may also involve microbial bioconversion processes such as fermentation and anaerobic digestion (AD) to enhance conversion efficiency. Third-generation biofuels are derived primarily from algae, while fourth-generation biofuels integrate genetic engineering and synthetic biology tools together with carbon capture strategies (Labbi 2017). However, the integration of bioenergy into thecontinent’s current energy mix faces challenges such as inefficient conversion pathways, weak policy frameworks, infrastructural limitations, and a relatively low presence in the global research landscape. These systemic barriers stem from limited investment by both the private sector and governments, particularly in developing countries where innovation remains poorly institutionalized. The absence of adequate incentives and the underdevelopment of double and triple-helix structures linking universities, research institutes, and the private sector constrain the emergence of functional innovation ecosystems. In such contexts, basic and applied research rarely translates into marketable bioenergy products, as the industrial actors required for technological and product development are either absent or insufficiently engaged. Funding scarcity compounds this challenge, limiting both the scaling of promising technologies and the retention of scientific expertise. In addition, the lack of region-specific life cycle assessments (LCA) and techno-economic assessment (TEA) models limits the ability to evaluate and scale bioenergy solutions effectively (Jain and Kumar 2024; Raina et al. 2024). Rather than treating these elements in isolation, there is a need to examine how biomass resources, conversion technologies, and sustainability metrics interact within an integrated system. This review examines biomass valorization and bioenergy systems in Africa, with an emphasis on resource availability, conversion technologies, and their integration within circular bioeconomy frameworks. Methodologically, the analysis combines quantitative and qualitative perspectives to capture both technological progress and broader system-level dynamics. Bibliometric and patent data were used to map research activity, identify dominant themes, and examine collaboration patterns across the continent. These insights also shaped the organization of the review, ensuring that biomass resources, bioconversion pathways, and LCA are discussed in a way that reflects current scientific and technological developments.

## Bibliometric and patent analysis

Bibliometric analysis is used to assess research trends and collaboration networks (Alao et al. 2024). Accordingly, the bibliometric and patent data used in this study were retrieved on March 3, 2026, from the Scopus and World Intellectual Property Organization (WIPO) PATENTSCOPE database, respectively. Both datasets were collected using the search query: TITLE-ABS-KEY (“biomass” AND (“bioethanol” OR “biogas” OR “biodiesel” OR “biohydrogen” OR “bioelectricity”)). The bibliometric dataset covers the period from 2010 to 2025 and includes articles, reviews, book chapters, and conference papers published in English. Retrieved records were screened to remove duplicates and to ensure relevance to bioenergy research. Mapping and network visualization were carried out using VOSviewer (version 1.6.20). Keyword co-occurrence networks, country-level collaboration maps, and thematic clusters were generated using the full-counting approach, with a minimum occurrence threshold of five keywords to capture dominant research themes and collaboration patterns. The patent records retrieved from WIPO PATENTSCOPE were further filtered to retain only patents directly related to bioenergy technologies. The International Patent Classification (IPC) system was employed to categorize patents and identify technological focus areas. IPC subclass frequencies were analyzed to determine dominant innovation domains, and country-level patent distributions were examined to assess geographic specialization in bioenergy technologies.

## Research trends, keyword co-occurrence, and collaboration networks

**Fig. 1:**
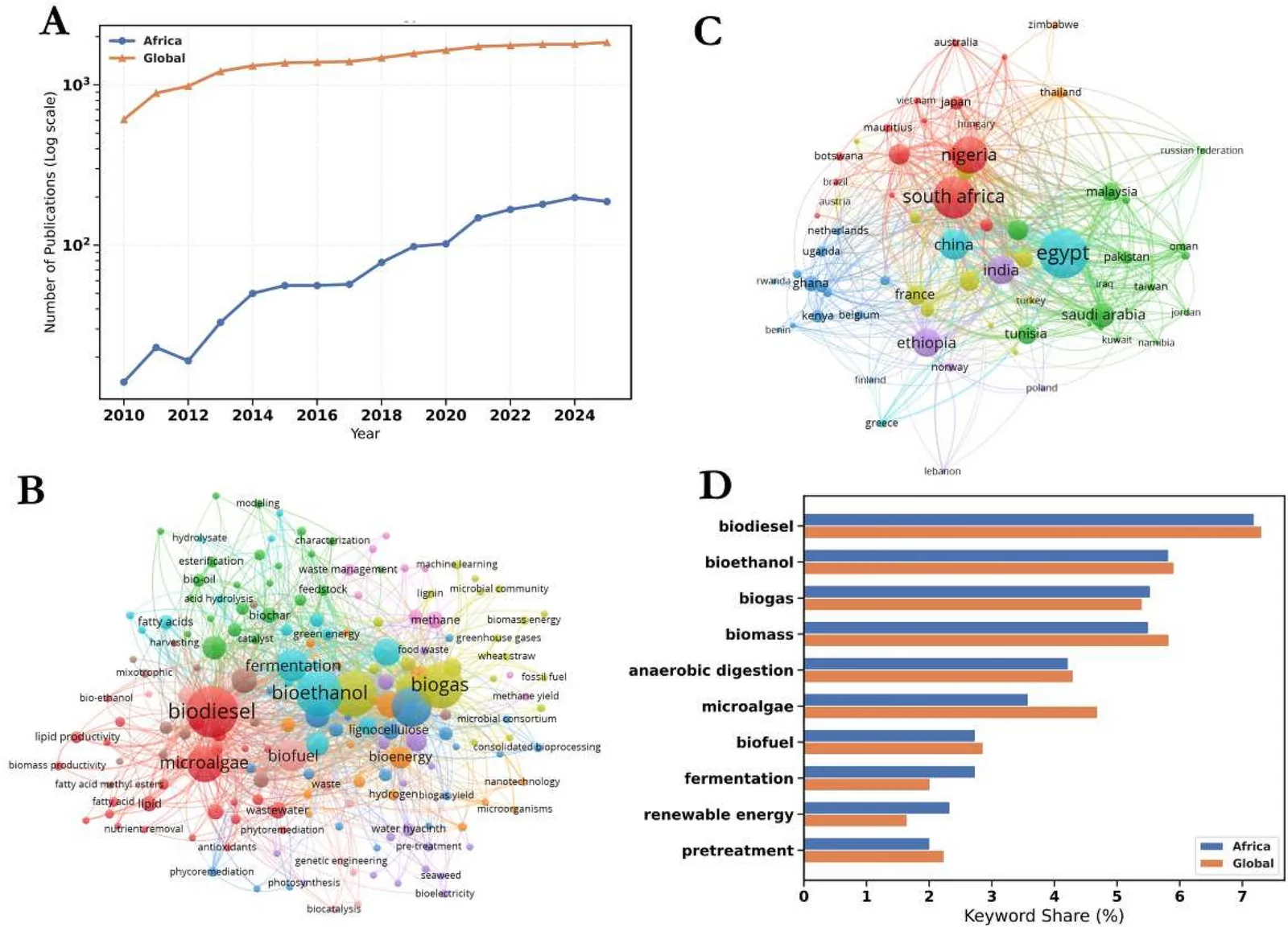
Bibliometric overview of bioenergy research: (A) Annual publication trends (2010-2025). (B) keyword co-occurrence network. (C) country collaboration network. (D) Relative comparison of keyword frequency (%) between African and global research outputs.

The annual publication trend between 2010 and 2025 is shown in Fig. 1A on a logarithmic scale. At the global level, research output shows sustained and nearly linear growth over the study period. This suggests the strategic prioritization of renewable energy technologies in response to climate change mitigation and energy security concerns. The publication trajectory shows no structural stagnation, indicating continued scientific investment in biomass-based energy systems. In comparison, Africa’s publication output remains lower in absolute terms but follows a steeper growth trajectory. However, the magnitude of the gap between Africa and the global average highlights structural disparities in research capacity, funding access, and technological infrastructure. The keyword co-occurrence network (Fig. 1B) highlights a highly interconnected thematic structure centered on biodiesel, bioethanol, biogas, fermentation and lignocellulose. The prominence of biodiesel and bioethanol indicates sustained interest in liquid biofuels as substitutes for fossil-based fuels. Simultaneously, emerging thematic clusters associated with microalgae, biohydrogen (BioH_2_), and consolidated bioprocessing suggested diversification into advanced biological pathways and non-conventional feedstocks. Fig. 1C further illustrates a structured collaboration landscape. South Africa, Egypt, Nigeria, and Ethiopia emerge as the most influential African nodes, as indicated by their larger node sizes and dense linkages. South Africa appears as a central hub, maintaining extensive connections with both intra-African partners and extra-continental collaborators. The network also shows strong linkages between African countries and major global research actors such as China, India, France, Malaysia, and Saudi Arabia. This suggests that research in Africa is significantly integrated into broader international collaboration frameworks. Finally, Fig. 1D compares keyword distribution between African and global research outputs. Both emphasize core topics such as biodiesel and bioethanol, with global research showing a stronger focus on emerging areas such as microalgae-based systems and advanced biofuel pathways.

### Patent trends, IPC classification, and geographic distribution

**Fig. 2:**
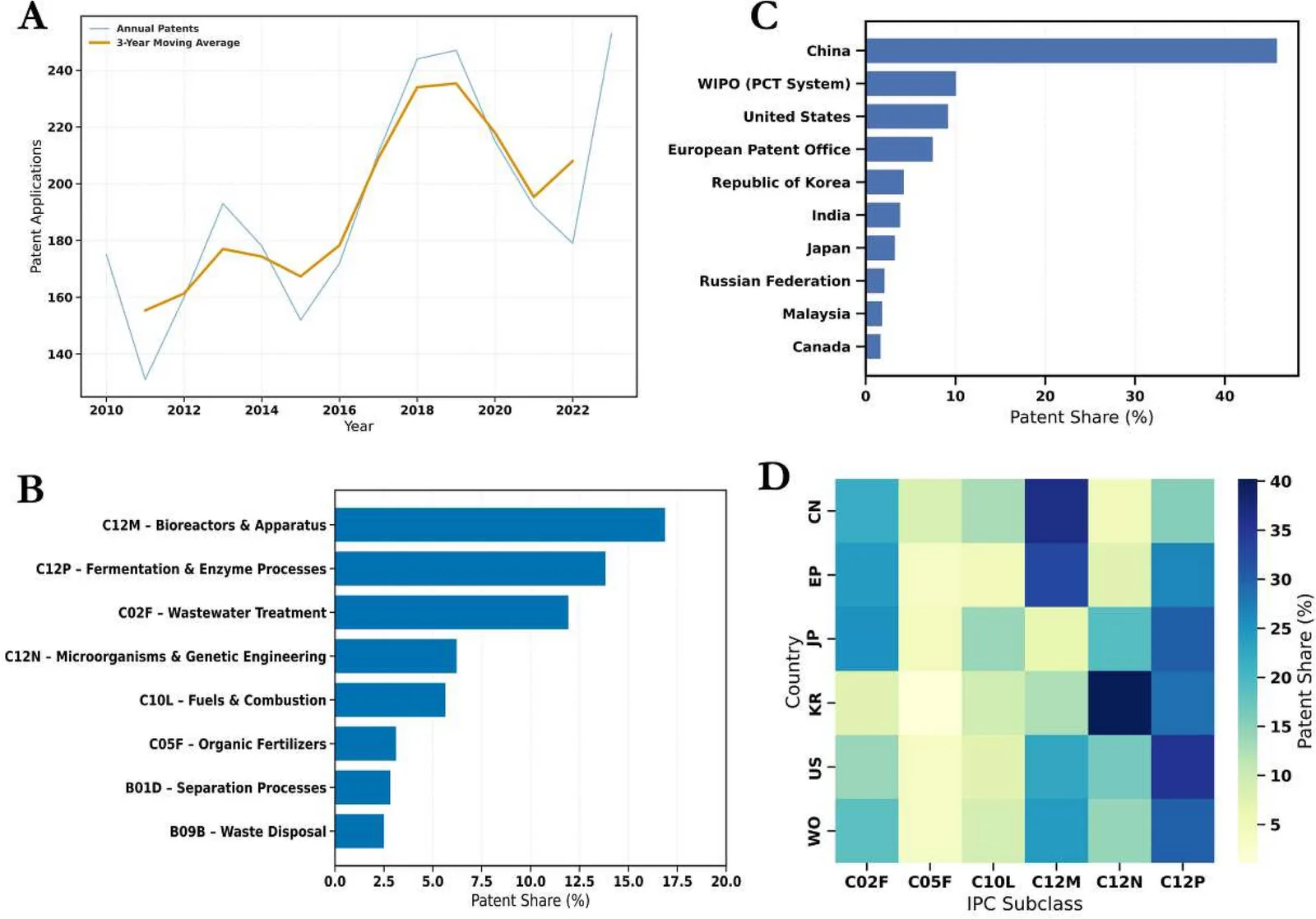
Patent landscape of biomass-to-bioenergy technologies, 2010–2023 (WIPO PATENTSCOPE). (A) Annual patent applications with a 3-year moving average. (B) Share of the eight leading IPC subclasses. (C) Share of the ten leading filing offices. (D) Patent share by IPC subclass for the six leading offices (CN, EP, JP, KR, US, WO).

Patent data were analyzed to gain insights into the geographic distribution of innovation in bioenergy systems. As shown in Fig. 2A, patent applications in bioenergy technologies show moderate growth, with a notable increase after 2018. The three-year moving average smooths fluctuations, illustrating a clear upward trend in bioenergy innovation. The IPC classification analysis (Fig. 2B) provides a standardized way of identifying the technological focus of patents (World Intellectual Property Organization 2022). The frequently occurring IPC codes include C12M (bioreactors and apparatus), and C12P (fermentation and enzymatic processes). This indicates a clear focus on biochemical conversion technologies such as fermentation, AD, and bioreactor systems. Additional classification codes include C02F (wastewater treatment) and C12N (microorganisms and genetic engineering) which reflect advances in the use of microorganisms in environmental remediation and bioenergy technologies. In terms of geographic distribution, Fig. 2C shows that China, WIPO, US, and the European Patent Office (EPO) lead global patent activity, indicating strong industrial and institutional engagement. Other countries such as India and Malaysia are also significant contributors, highlighting the growing interest of emerging economies. African nations remain largely underrepresented in global bioenergy patenting. This underrepresentation is more accurately explained by structural barriers than by technological immaturity: the absence of supportive public policies, the scarcity of dedicated funding mechanisms, and the lack of coordinated national innovation strategies limit patent activity across the continent. Bioenergy conversion technologies are, by now, well-established globally; the gap in African patenting therefore reflects an innovation ecosystem deficit rather than a knowledge deficit. Closing this gap requires deliberate investment in research infrastructure, technology transfer mechanisms, and policy frameworks that incentivize industrial application of scientific outputs. Regarding the collaboration network, the country/IPC specialization heatmap (Fig. 2D) further highlights clear technological differentiation among leading patenting countries. China shows a strong concentration in C12M, highlighting dominance in process engineering and reactor technologies. The United States and WIPO filings exhibit high shares in C12P, indicating an emphasis on biochemical conversion pathways. The EPO demonstrates a more balanced distribution across subclasses, particularly in C12M and C02F. he Republic of Korea also shows marked specialization in C12N, suggesting advanced biological innovation capabilities. These patterns show that bioenergy patenting is heavily concentrated in biochemical conversion technologies, with distinct national specialization patterns reflecting strategic innovation priorities.

## Biomass as a renewable energy source in Africa

**Fig. 3:**
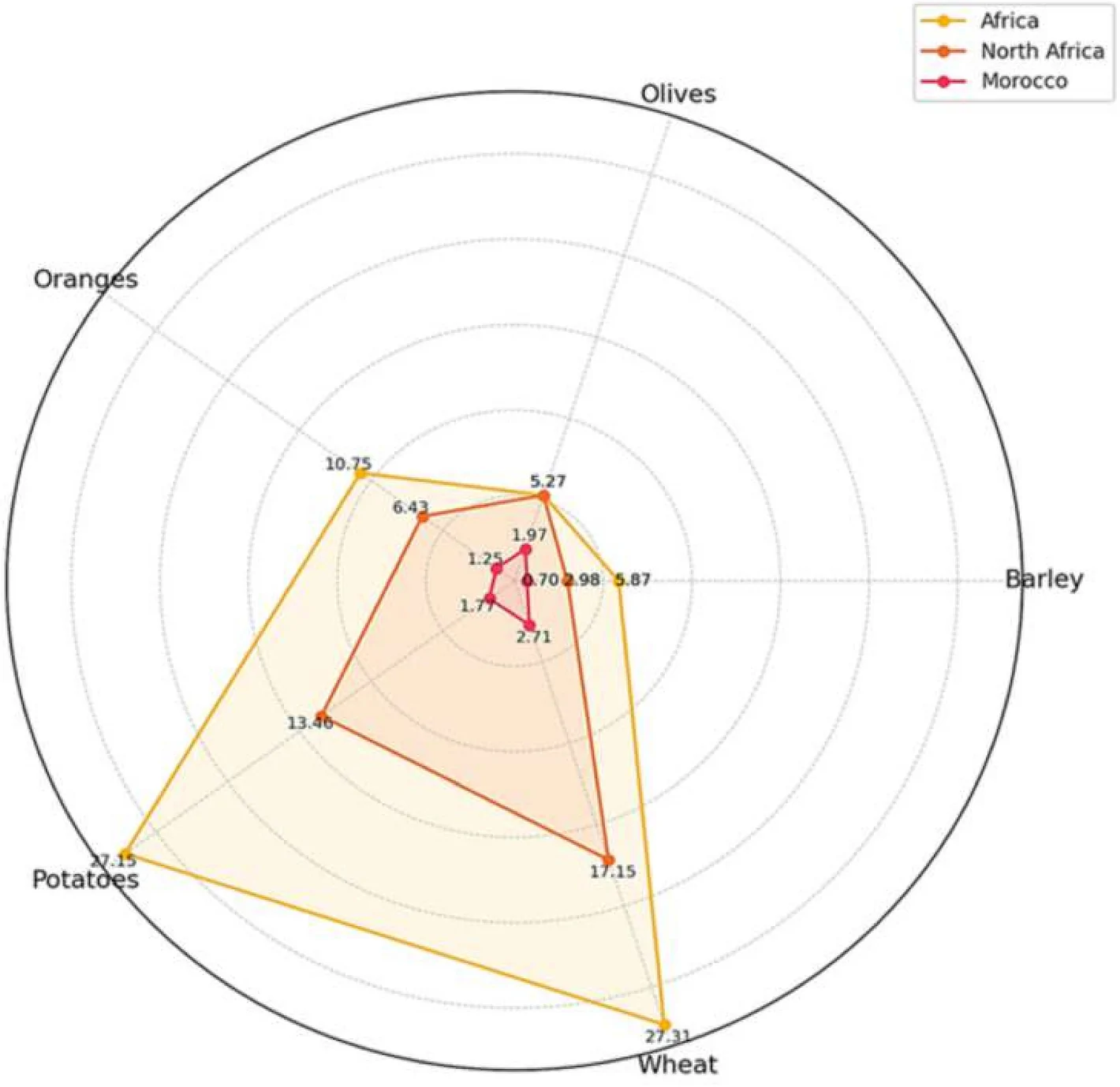
Production (million tonnes) of barley, olives, oranges, potatoes and wheat in Africa, North Africa and Morocco (Source: FAOSTAT 2024).

Biomass represents a key opportunity for renewable energy, sustainability and socio-economic development in Africa, where access to modern energy services remains uneven (Mensah and Yankson 2025). The continent is rich in biomass resources such as crops, forestry products, palm oil by-products, and livestock waste, which significant bioenergy potential (Chowdhury et al. 2025; Weldesemayat Sileshi et al. 2025). Although biomass is the primary feedstock for bioenergy (Dos Santos et al. 2025), there is still unequal access to energy across the continent, and people still rely on traditional biomass as a source of fuel for cooking (IRENA 2024). This demonstrates the urgent need for efficient biomass conversion technologies and clean energy solutions, particularly in rural areas still dependent on traditional methods. Fig. 3 compares the production of barley, olives, oranges, potatoes, and wheat across Africa, North Africa, and Morocco, highlighting the significant contribution of these crops to regional biomass availability.

### Classification and major sources of biomass in Africa

Biomass resources in Africa can be broadly classified into agricultural residues, forest biomass, energy crops, and algal biomass. These categories represent diverse and complementary feedstocks with significant potential for renewable bioenergy production and bioproduct development. Agricultural residues are among the most accessible and low-cost biomass sources for energy and bioproducts (Balasubramani et al. 2023). Forest operations generate large quantities of residues such as branches, bark, sawdust, wood chips, and logging residues, all of which can contribute to bioenergy production, pulp and paper manufacturing and soil amendment. In 2025, Africa had approximately 635 million hectares of forested land, covering 21% of the continent’s total area and representing a significant source of biomass with potential use as a energy resource (Kelatwang and Garzuglia 2006). However, in Morocco, where forests cover about 9 million hectares (about 5.8 million hectares of forest formations and 3.2 million hectares of alfa-grass steppe), this resource remains underexploited despite its significant energy potential (Naimi et al. 2017). A study by Bendaoud et al. (2022) in the Fez-Meknes region of Morocco found that solid waste from forests constitutes a major portion of green waste, with high cellulose content suitable for energy production. Dedicated energy crops also serve as another promising biomass source. These are plant species specifically cultivated for energy generation (Devi et al. 2022). According to Singh et al. (2023), energy crops can be grown on marginal or degraded lands, thereby avoiding competition with food production and reducing potential land-use conflicts, which is a major drawback of first-generation biomass. The use of non-food or conventional crops for biofuel production presents several environmental benefits, such as reducing GHG emissions and enhancing soil fertility by preventing land degradation and protecting water quality (Dida 2024). Africa currently holds the greatest potential for global energy crop production and supply (Dahunsi et al. 2020), positioning the continent as an increasingly attractive destination for international investment in the bioenergy sector. Finally, algal biomass is emerging as a next generation feedstock for bioenergy production. Microalgae, in particular, are characterized by rapid growth, adaptability to diverse environments, and the ability to capture carbon dioxide (CO_2_), making them valuable for sustainable fuel production (Encarnação et al. 2023). In the African and specifically Moroccan context, microalgae research has gained momentum, though it remains at an early applied stage. Morocco’s Atlantic coastline and warm climate provide favorable conditions for microalgal cultivation, and Spirulina is currently the only species cultivated at scale in the country, primarily for food and nutraceutical markets. ( El Arroussi et al. 2017) Macroalgal biomass occurs abundantly along West African coasts and has also attracted regional interest as a fermentable substrate for biogas and bioethanol production (Ndiaye et al. 2026), with Morocco’s macroalgal diversity (over 600 documented species) representing an underutilized bioenergy resource. From a biochemical standpoint, lipid-rich microalgal species such as *Nannochloropsis* and *Botryococcus braunii* are suitable for biodiesel production, while carbohydrate-rich species such as *Chlorella* and *Spirulina* are ideal for bioethanol production (Medeiros et al. 2024). Additionally, protein-rich microalgae such as *Chlorella* and *Scenedesmus* are being studied for biogas production through AD (Anacleto et al. 2025). In addition to conventional biofuels, algae can support the production of advanced forms of bioenergy, such as BioH_2_ through photobiological processes and dark fermentation, and bioelectricity through microbial fuel cells (MFCs)(González Fernández et al. 2025; Pengadeth et al. 2024). However, the commercialization and large scale harvesting of algal biomass face challenges such as high costs, limited scalability, and energy-intensive harvesting (Sarwer et al. 2022). Ongoing advances in cultivation, genetic engineering, and innovative recovery techniques like electro-flocculation and flocculation-assisted centrifugation are improving biomass yield and economic viability (Paul et al. 2025).

### Case study: biomass resource potential and availability in Morocco

Africa's abundant agricultural residues and organic wastes, together with dedicated energy crops, provide a promising opportunities for renewable energy development (Fertahi et al. 2023). Traditional biomass is mainly used for cooking, a highly inefficient practice (10–20% efficiency) associated with serious risks, with 500 000 annual deaths globally linked to indoor air pollution (Urban et al. 2025). Morocco has considerable biomass potential, derived primarily from agricultural activities, domestic waste streams, and marine biomass. It is estimated that approximately 23 million tons of crop residues are generated annually, of which less than 2% are currently used for bioenergy generation (Mahdavi et al. 2023). This reflects a major gap between potential and sustainable utilization. The country’s agricultural sector is dominated by olive, citrus, and cereal production, which generates large quantities of biomass. As one of Africa’s leading olive producers, Morocco generates considerable amounts of olive pomace, which is already used locally as a heat source. This by-product also holds additional opportunities for valorization into biogas, biochar, and other value-added products, supporting both energy diversification and circular bioeconomy strategies (Fertahi et al. 2023). Citrus processing residues further contribute to the biomass pool, with an estimated annual potential of approximately 38.7 thousand tons (Labbi 2017). In addition to terrestrial resources, Morocco’s extensive coastline provides access to significant marine biomass. The country hosts approximately 600 algal species, of which more than 150 are considered to have high economic value (Fertahi et al. 2023). A total of 22 and 21 macroalgal species have been reported from the Sidi Rahal region and the southern Atlantic coast of Morocco (Tarfaya to Laayoune), respectively (El Meraoui et al. 2024; Boutafda et al. 2025). Moreover, the southern coast offers optimal conditions for large-scale algae cultivation using raceway pond systems (Wahby et al. 2018). However, algae’s bioenergy potential remains underexploited due to challenges such as storage instability, rapid biological degradation, and limited cultivation strategies. According to IRENA (2024), the annual net primary production of biomass in Morocco is estimated to be 1.5 tons of carbon per hectare per year, which is below the global average. Nevertheless, the combination of terrestrial and marine biomass resources offers Morocco a strategic opportunity to boost renewable energy.

## Current status of bioenergy in Africa

**Fig. 4:**
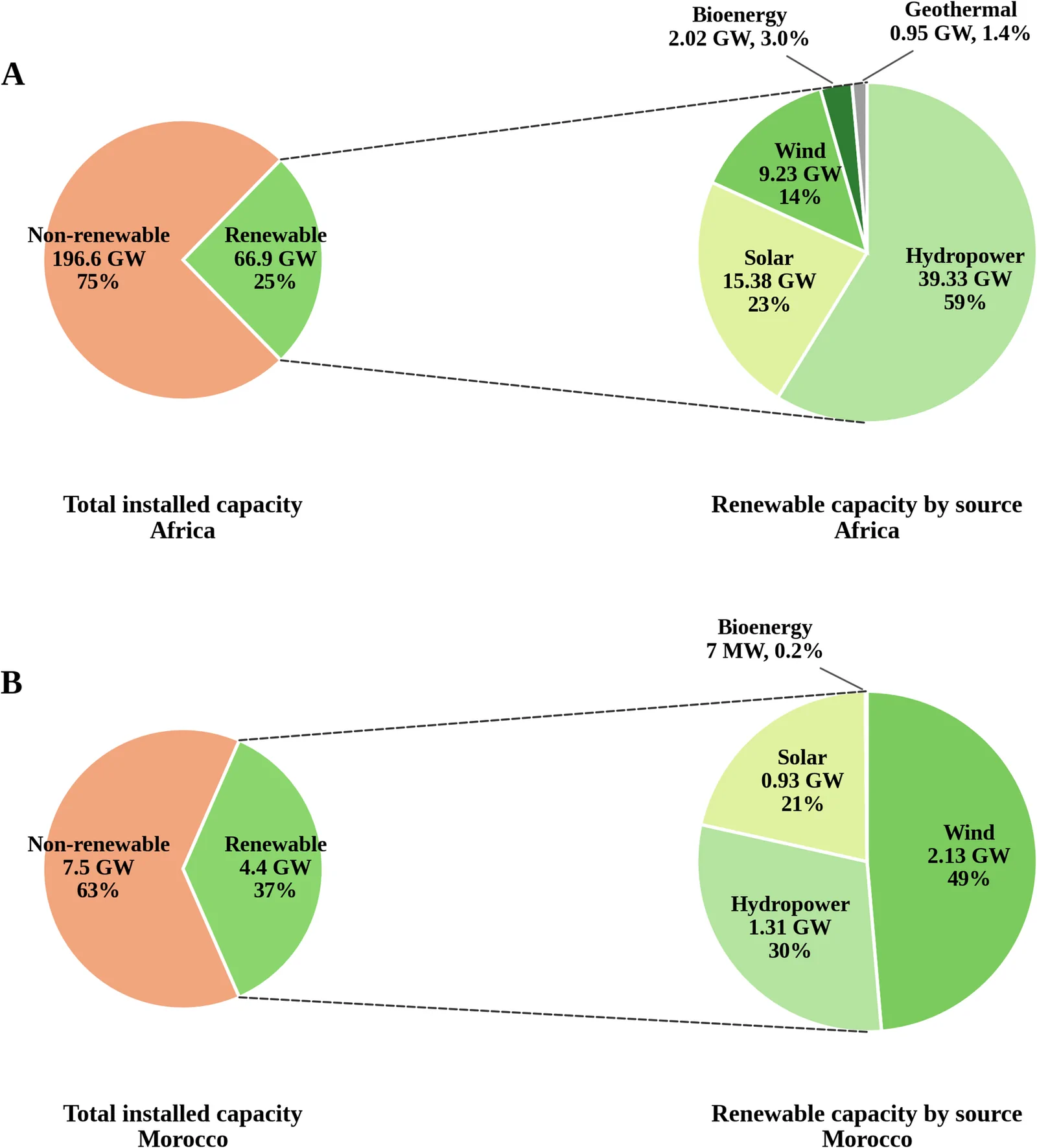
Installed electricity capacity in (A) Africa and (B) Morocco at the end of 2024, showing the renewable versus non-renewable split (left) and renewable capacity by technology (right) (IRENA, 2024).

Global demand for biofuels continues to rise, highlighting their growing role in the transition toward low-carbon energy systems. According to the International Energy Agency (IEA 2023), biofuel demand is expected to increase at an average rate of approximately 28% between 2021 and 2026 . This will increase demand to 186 billion liters by 2026, representing 41 billion liters more than in 2020 (IEA 2023). Over the longer term, biofuels are expected to play a significant role in decarbonizing the transport sector, with the potential to reduce GHG emissions by more than 80% relative to fossil fuels (Suhalka 2024). In Africa, the contribution of bioenergy to the overall energy mix remains limited. Bioenergy accounts for approximately 3% of Africa’s total installed renewable power capacity (Fig. 4A). This share lags behind comparable developing regions: bioenergy contributes roughly 8–10% of renewable capacity in Latin America and exceeds 10% in several South and Southeast Asian countries. Within Africa, installed biopower capacity is unevenly distributed, with most countries exhibiting negligible modern bioenergy deployment. By comparison, solar energy accounted for approximately 23% of the continent’s renewable power capacity in 2023, corresponding to 15.5 GW. . Significant bioenergy investments have been made across the continent, especially in North Africa, which received USD 17.5 billion, between 2010 and 2020, representing 32% of Africa’s total renewable energy investment, mainly in Morocco and Egypt (IRENA 2024). Countries are increasingly utilizing agricultural residues, forest by-products, and organic waste to produce biogas, bioethanol, and biofuels, supporting energy security and emission reduction goals (Same et al. 2024; Leal Silva et al. 2025) Morocco, which is heavily dependent on energy imports (90–95%), has prioritized renewable energy, including bioenergy, to reduce reliance on foreign hydrocarbons (Laaroussi and Bouayad 2020; US. Department of Commerce 2024). In the mid-2010s, the industrial sector was Morocco’s largest electricity consumer (43.6%), followed by households (32.8%), and agriculture/commercial uses (22.4%) (Labbi 2017). This distribution highlights the significant energy demand from productive sectors, indicating the importance of diversified and reliable energy sources.

## Bioenergy and production pathways

 Africa has substantial bioenergy potential; however, its effective exploitation remains constrained by a common challenge across all conversion pathways: variability in feedstock availability and quality.The composition of lignocellulosic biomass (cellulose crystallinity, lignin content, moisture fraction, and ash load) fluctuates significantly with plant species, harvest season, storage conditions, and geographic origin, and these fluctuations propagate directly into conversion efficiency, process stability, and energy yield. High lignin content reduces enzyme accessibility to cellulose and increases hydrolysis time; elevated moisture reduces calorific value and raises pre-treatment energy demand; silica-rich ash from residues such as rice husk causes reactor scaling and inhibitor accumulation; and in AD systems, an imbalanced carbon-to-nitrogen ratio triggers either ammonia toxicity or volatile fatty acids (VFAs) accumulation that destabilizes methanogenesis (Chowdhury et al. 2025). For biodiesel, lipid profile variability directly affects fatty acid methyl esters (FAME) cold-flow properties and oxidative stability. These are not incidental engineering challenges but rather the primary reason why technologies that perform well at laboratory scale on standardized substrates fail to replicate that performance on the heterogeneous agricultural residues that dominate Africa’s actual supply. Feedstock characterization and pre-treatment standardization are therefore prerequisites for any reliable, scalable bioconversion system, regardless of which pathway is chosen (Kumar et al. 2023). Because feedstock heterogeneity represents a common bottleneck, pretreatment is a decisive operation governing downstream yields, and its selection must be matched to both the substrate and the target product. Physical methods, including milling, extrusion, and steam explosion, reduce particle size and may decrease cellulose crystallinity. Chemical methods target specific structural components: dilute-acid hydrolysis solubilizes hemicellulose and exposes cellulose, alkaline treatment using NaOH or lime cleaves lignin–carbohydrate linkages, while organosolv and ionic-liquid systems can achieve extensive delignification but generally involve higher reagent and process costs. Biological pretreatment, based on ligninolytic fungi or enzyme cocktails, offers a lower-energy alternative, although it is typically slower (Nair and Verma 2025; Kumar et al. 2026). No single pretreatment method is universally optimal because its effectiveness depends on feedstock characteristics, conversion objectives, energy requirements, inhibitor formation, and process economics. Lignin is increasingly regarded not as a disposal problem but as a potentially valuable co-product because its conversion into aromatic chemicals can improve overall biorefinery economics (Jiju et al. 2025). Accordingly, aligning pretreatment severity with feedstock composition and product specifications is one of the most important determinants of process viability.

### Integrated bioprocesses for bioethanol production

**Fig. 5:**
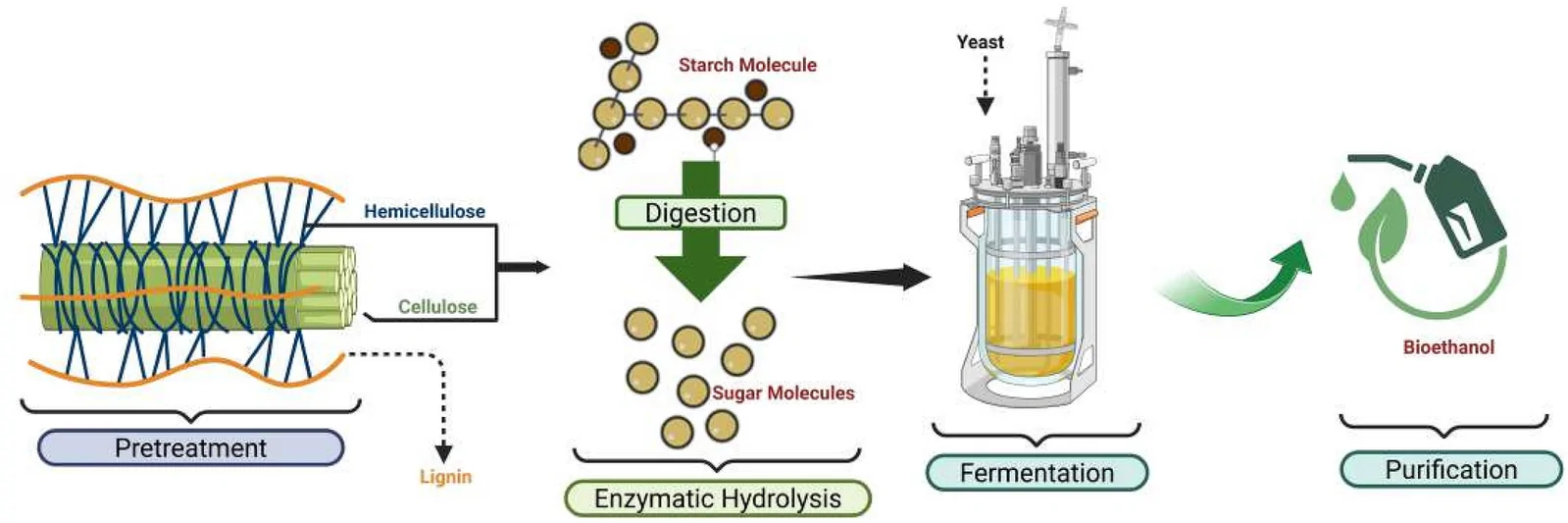
Bioethanol production from lignocellulosic biomass: pretreatment, enzymatic hydrolysis, fermentation and ethanol recovery.

Global bioethanol production reached approximately 130 billion liters in 2022, with over 70% originating from the United States and Brazil, while Africa remains a marginal producer despite theoretical feedstock potential (Same et al. 2024). South Africa has introduced ethanol-blending mandates, and Uganda, Côte d'Ivoire, Nigeria, Ghana, and Cameroon are expanding production from agricultural residues (Same et al. 2024; Leal Silva et al. 2025). The barriers are structural rather than technological: limited pre-treatment infrastructure, high enzyme costs, land competition with food crops, and the absence of stable investment frameworks. Depending on feedstock and pathway, bioethanol reduces GHG emissions by 30–80% and particulate emissions by up to 50% relative to gasoline (Jain and Kumar 2024). The key performance limitation in lignocellulosic bioethanol is not the well-established biochemical sequence of pre-treatment, saccharification, and fermentation, but enzyme cost reduction and configuration optimization (Fig. 5). Enzyme recycling *via* ultrafiltration and immobilization on nanostructured supports now enables multiple reuse cycles, substantially lowering enzyme loading requirements and improving process economics (Meena et al. 2021; Hussain et al. 2024). On the fermentation side, hybrid hydrolysis and fermentation (HHF) outperforms both separate hydrolysis and fermentation (SHF) and simultaneous saccharification and fermentation (SSF) configurations, achieving up to 48.5 g/L ethanol against 12.4–32.3 g/L under comparable conditions (Wang et al. 2025). Consolidated bioprocessing (CBP), in which enzyme production, hydrolysis, and fermentation occur within a single engineered microorganism, remains the most capital-efficient configuration on the horizon, with *Saccharomyces cerevisiae* and *Clostridium* species engineered *via* CRISPR-Cas9 showing extended pentose sugar utilization and inhibitor tolerance (Periyasamy et al. 2023; Aggarwal et al. 2023). Purification is constrained by the ethanol–water azeotrope at 95.6 wt %; molecular sieve dehydration is the established industrial solution, while membrane pervaporation offers a more energy-efficient and chemically cleaner emerging alternative (Peng et al. 2021; Zafarnak and Rahimpour 2024). In Morocco, enzymatic saccharification has been investigated olive pomace, cereal straw, and date palm rachis. These are feedstocks that are abundant, locally generated, and largely unprocessed for energy recovery. Several studies have characterized the saccharification potential of these substrates and explored their suitability as second-generation bioethanol feedstocks. Nait M’Barek et al. (2020) demonstrated CBP of olive mill waste using indigenous wood-decay fungi isolated from central Morocco, achieving competitive bioconversion yields from one of the country's most recalcitrant agro-industrial waste streams. Morocco's citrus processing sector in the Souss-Massa region generates approximately 38,700 tons of processing residues annually (Labbi 2017). The lignocellulosic fraction remaining after industrial pectin extraction is a near-term second-generation bioethanol substrate once laboratory-validated pre-treatment protocols are transferred to pilot scale.

### Biomass-to-biodiesel conversion processes

Global biodiesel production is led by the EU, Indonesia, and Brazil, with a gradual shift away from first-generation food-crop feedstocks toward waste-derived oils and microalgal lipids (EUROPEAN BIODIESEL BOARD STATISTICAL REPORT 2023b). The dominant conversion pathway is base-catalyzed transesterification of triglycerides with methanol, producing FAME and glycerol as a co-product. Process efficiency is governed by the alcohol-to-oil molar ratio, catalyst concentration, and reaction temperature. Biochar-based heterogeneous catalysts are emerging as technically and economically viable alternatives to conventional NaOH/KOH: thermally activated biochar from *Camellia japonica* has achieved FAME yields of 92.4 wt% at temperatures above 240 °C, avoiding the catalyst separation difficulties and glycerol contamination inherent to homogeneous systems (Park et al. 2025). lipid profile variability, the principal feedstock challenge for biodiesel is the fatty acid chain length distribution, which directly affects FAME cold-flow properties and oxidative stability. This requires either feedstock blending or post-processing additive strategies to meet fuel standards. Olive pomace in Morocco contains residual lipids that can be recovered by hexane extraction, a process already practiced industrially in the Meknes-Fez and Marrakech-Safi regions. directly transesterified , generating glycerol as a co-product. Microalgal oils from lipid-rich strains such as *Nannochloropsis* and *Botryococcus braunii* under investigation at several institutions, particularly in relation to resources along Morocco's Atlantic coastline. This approach represents a third-generation feedstock pathway requiring the scale-up of cultivation and low-energy harvesting technology before commercial viability can be achieved (Laaroussi et al 2020).

### Anaerobic digestion for biogas production

**Fig. 6:**
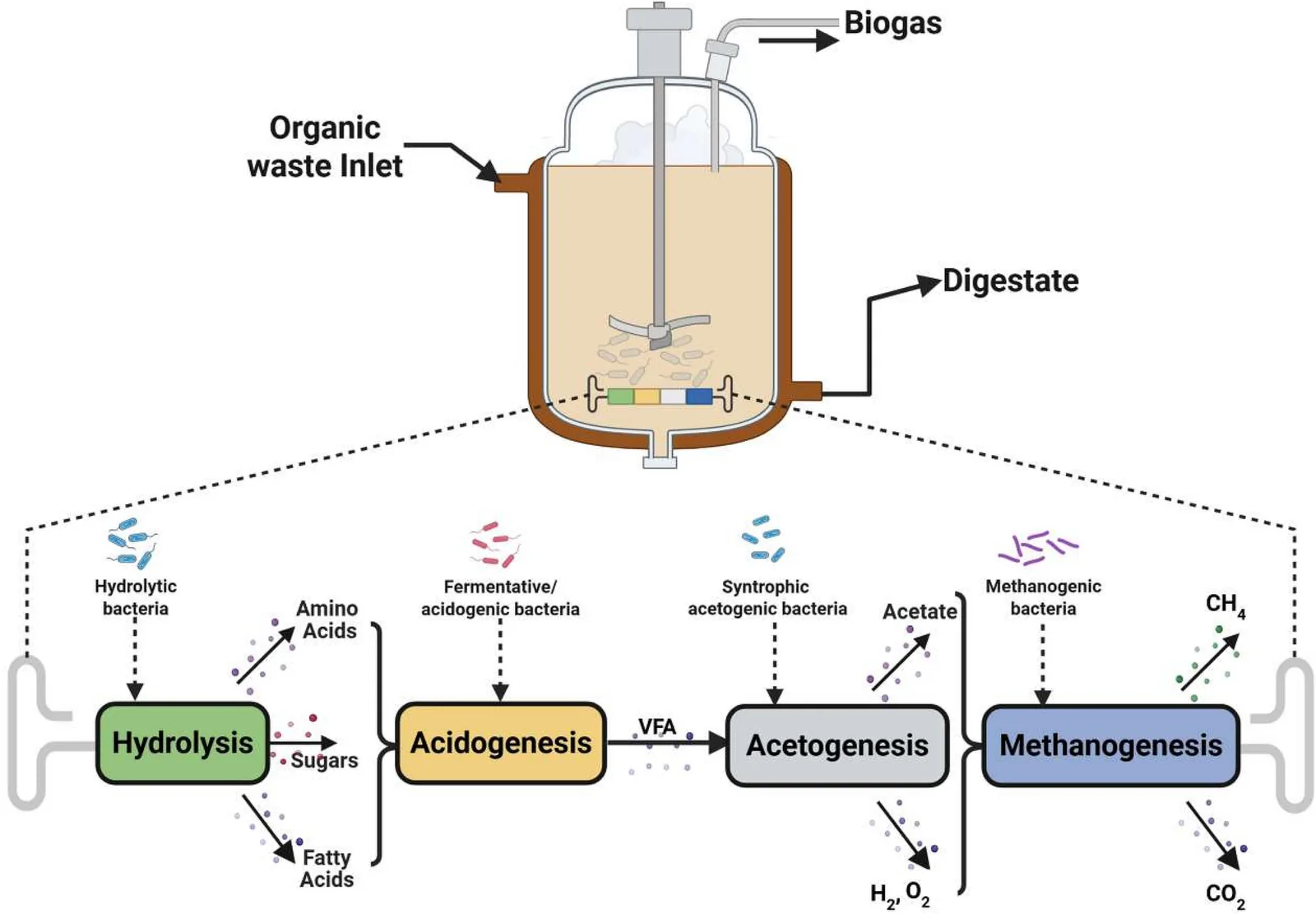
Anaerobic digestion of organic waste to biogas: digester layout and the four stages of hydrolysis, acidogenesis, acetogenesis and methanogenesis

AD is one of the primary biological pathways for the energy recovery of residual organic matter. It relates on a coordinated sequence of four microbial stages—hydrolysis, acidogenesis, acetogenesis, and methanogenesis—resulting in the production of biogas composed primarily CH_4_ and CO_2_(Fig. 6) (Niya et al. 2026). At the operational level, AD performance depends critically on the feedstock C/N ratio, organic loading rate, and temperature regime. Imbalanced C/N ratios accelerate either ammonia toxicity or VFAs accumulation, both of which destabilize methanogenesis (Lohani and Havukainen 2018). Consequently, controlling these parameters remains essential for ensuring stable and efficient methane production. However, recent research goes beyond the mere optimization of operating conditions and is increasingly focusing on microbial community engineering. For instance, inoculation with enriched microbial consortia can more than double the biogas yield obtained from complex lignocellulosic substrates, the degradation of which is generally limited in systems relying on a single microbial strain 

(Niya et al. 2024). Similarly, the addition of magnetic nanoparticles can facilitate direct electron transfer between microbial species and enhance syntrophic interactions within anaerobic biofilms, thereby accelerating substrate degradation and methane production

 (Dar et al. 2025). In parallel, metagenomic approaches offer the possibility of characterizing the communities present, diagnosing microbial imbalances and designing targeted interventions to restore digester performance. These advances thus mark a gradual shift from empirical process management to data-driven microbial engineering. (Niya et al. 2024b). Beyond energy production, AD also yields a digestate rich in organic matter and nutrients that can be used as a biofertilizer. Its agricultural application helps partially reduce reliance on synthetic fertilizers, replenish soil nutrients, and enhance the circularity of production systems (Karouach et al., 2024). This synergy between bioenergy production and nutrient recycling is of particular strategic importance to Morocco, given the significant role agriculture and the phosphate industry play in its economy.

Despite these advantages and technological progress, the deployment of biogas in Africa remains uneven and technically fragile. Around 40% of domestic digesters installed on the continent have become non-operational, mainly due to insufficient maintenance infrastructure, lack of technical skills and the absence of sustainable support for users (Fertahi et al., 2023). This situation shows that the availability of technology is not enough to guarantee its sustainability and that its adoption must be accompanied by appropriate training, maintenance and financing systems.

 In Morocco, annual biogas production is estimated at around 21.6 million m³. It is mainly based on wastewater treatment plants operated by the National Office of Electricity and Drinking Water (ONEE), as well as on the recovery of landfill gas at the Médiouna site, near Casablanca (Fertahi et al. 2023).However, this level of production remains limited given the large quantity of organic residues generated by the country's agricultural and agro-industrial activities. Among these resources, the by-products of the olive industry represent a particularly relevant source due to their seasonal abundance and their high organic load. At the level of the national olive oil sector, the AD of olive mill wastewater could contribute to the decentralized production of heat and electricity for rural communities in key olive-growing regions. The availability of this biomass—concentrated between October and January—would make it possible to meet seasonal energy needs while reducing the environmental impacts associated with storing the wastewater in open-air ponds (Sikaoui et al. 2024).Their recovery by AD thus constitutes an integrated approach combining production of renewable energy, treatment of agro-industrial effluents and reduction of polluting emissions. AD could also be integrated with other technologies to ensure more complete valorization of olive by-products. While the liquid fractions, in particular the vegetable waters, can be directed towards the production of biogas, the solid pomace can be treated by slow pyrolysis in order to simultaneously produce biochar and syngas. The biochar obtained constitutes a carbon amendment capable of improving soil properties and complementing national fertilization strategies. Such complementarity between biological and thermochemical processes thus offers the prospect of an integrated biorefinery adapted to the Moroccan olive sector.Realizing this potential nevertheless requires the establishment of a favorable regulatory, technical and financial environment. It requires in particular the reclassification of vegetable oils, so that they are considered as a valuable resource rather than a simple polluting effluent, as well as the development of efficient reactors in the main olive-growing regions. Finally, given the fragmented structure of the sector and the predominance of small crushing units, cooperative models of collection, treatment and financing appear essential to pool investments and ensure the economic viability of the installations.

### Biological hydrogen production pathways from biomass

BioH_2_ production from biomass offers a low-carbon pathway distinct from steam methane reforming (SMR), which currently accounts for approximately 80–85% of global H_2_ supply but remains a carbon-intensive process that poses significant challenges to achieving global decarbonization targets (Ou-douaou et al. 2026). Two fermentative pathways are sufficiently established for comparative assessment: dark fermentation, in which anaerobic bacteria convert organic compounds into H_2_, CO_2_, and VFAs ; and photofermentation, in which purple non-sulfur bacteria utilize these organic acids to produce additional H_2_ under illumination. Integrated dark-photofermentation systems can substantially outperform either route alone. For instance, combined processes reached 270 mL H_2_/g substrate from wheat grain, compared with 170 mL/g from photofermentation alone (Das and Basak 2025). Microbial electrolysis cells (MECs) provide an electrochemically assisted route in which electroactive microorganisms oxidize biomass-derived substrates at the anode, while a low applied voltage drives H_2_ evolution at the cathode. H_2_ yield from pre-fermented food waste reached 1.029 L/g COD, corresponding to a 39.1% increase relative to the raw feedstock (Kim et al. 2025). In addition, dark fermentation–MEC integration achieves a H_2_ production rate of up to 3.2 L H_2_/L/d (Hussien et al. 2025). Scale-up constraints across all these routes include electrode cost, feedstock variability, and the need for robust process control under field conditions. Morocco has initiated waste to research covering both fermentative and MEC pathways (Ou-douaou et al. 2026). This marks an early contribution to BioH_2_ research within the North African scientific landscape. The MEC route is particularly promising in the Moroccan context because the low applied voltage required (typically 0.2–0.8 V) could be supplied at competitive cost from the country’s solar photovoltaic infrastructure, creating a direct synergy between the country’s growing renewable electricity sector and its emerging BioH_2_ activities. Country-specific techno-economic assessments of this integration are currently lacking in the literature, representing a clear research priority.

### Microbial fuel cell-based bioelectricity production from biomass

**Fig. 7:**
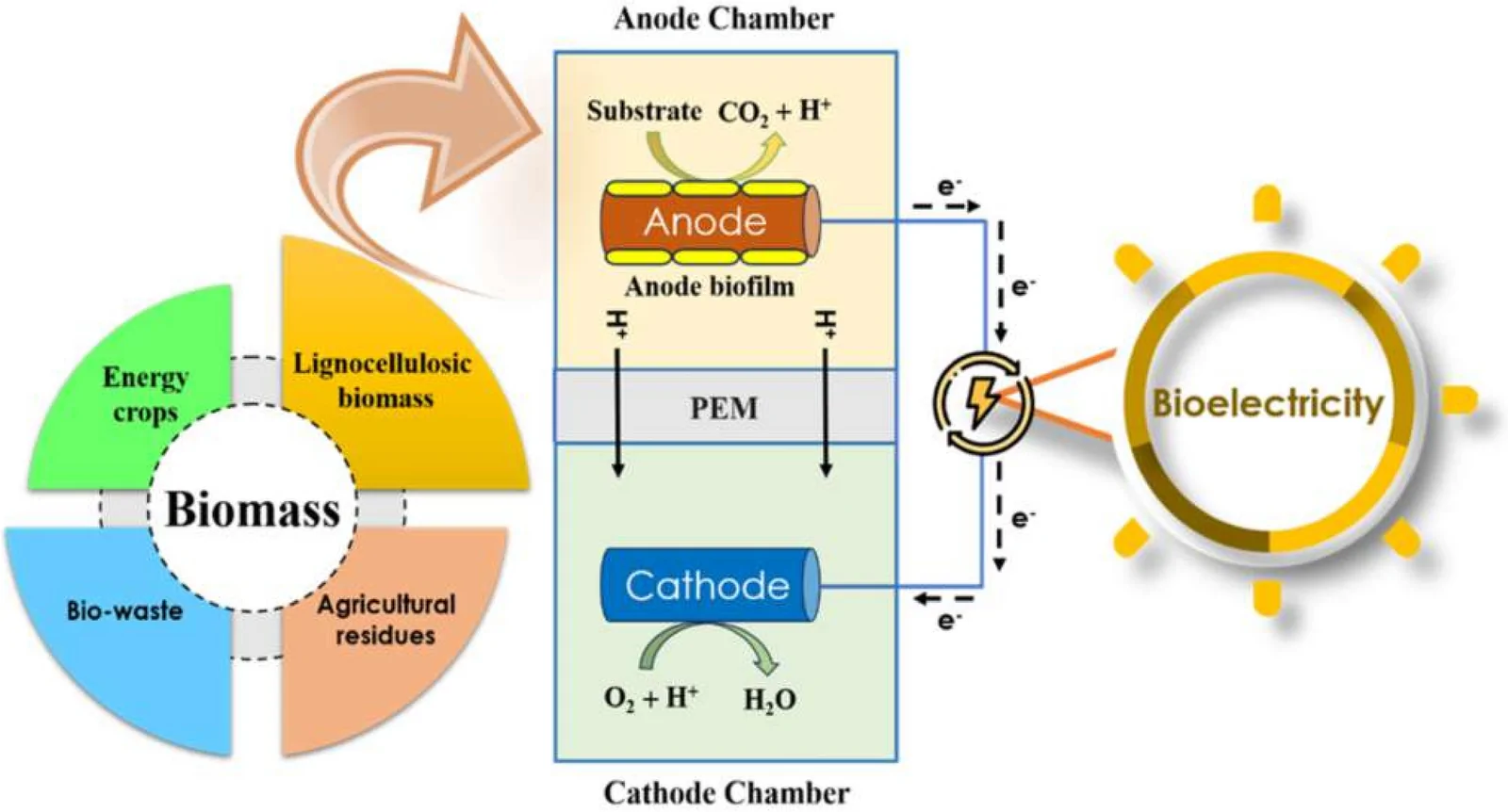
Dual-chamber microbial fuel cell converting biomass-derived substrates into bioelectricity.

MFCs convert the chemical energy of organic substrates directly into electricity through the metabolic activity of electroactive microorganisms, which oxidize organic matter at the anode and transfer the resulting electrons to the cathode *via* an external circuit (Fig. 7) (Musa et al. 2026). Lignocellulosic materials and algal biomass are the most studied feedstocks. Macroalgae, specifically *Laminaria* species, have also been successfully employed in MFC systems Zhang. (2018), highlighting the potential of Morocco’s Atlantic macroalgal diversity, comprising more than 600 documented species (Fertahi et al. 2023; Boutafda et al. 2025), as a largely unexplored feedstock resource for MFC applications. MFCs remain pre-commercial, as their near-term value in the African context lies in simultaneously treating high-strength organic effluents, including olive mill wastewater and agro-food processing streams, while recovering usable energy, potentially reducing the combined costs of waste management and energy supply in settings where both are constrained. To illustrate the diversity of bioconversion pathways and their effectiveness in converting biomass into bioethanol, biogas, BioH_2_, and bioelectricity, Table 1 presents selected case studies covering feedstock types, process technologies, microbial strains, operating conditions, and product yields. This offers valuable insights and benchmarks for assessing the efficiency, scalability, and sustainability of biorefinery processes.

**Table 1:** Overview of selected bioconversion case studies for bioethanol, biogas, biohydrogen, and bioelectricity production

Bioenergy type	Feedstock	Process	Microorganism/Catalyst	Operating conditions	Yield	References
Bioethanol	Sugar beet pulp (SBP)	SHF	SternEnzymeC21032 + Cellic C Tec2 / *S. cerevisiae *CY3079	28° C; pH 5; 72 h under anaerobic condition	44.26 g/L	Zohri et al. (2022)
	Pomegranate peel	SSF	Cellulase / *S. cerevisiae*	10% (v/v) of inoculum; 24 h; 120 rpm; anaerobic conditions	12.9 g/L	Mazaheri et al. (2021)
	Sugarcane bagasse	SSCF	Thermotolerant *S. cerevisiae*	120 h; 250 rpm; pH 5.5	36.0 g/L	Tu et al. (2021)
	*Phragmites australis*	HHF	*S. cerevisiae*	30 °C; 150 rpm; 72 h	48.5 g/L	Wang et al. (2025)
	Olive mill waste (OMW)	CBP	Wood-decay fungi	6.25% (v/v) inoculation ratio; 25° C	2.47 g/L	Nait M’Barek et al. (2020)
Biogas	Corn stalks + cow manure	AD	EM-4 microbial consortium	Ambient temperature	215.77 L/kg	Sumardiono et al. (2022)
	Corn stover		Rumen microbial community	Mesophilic (44°C); CSTR; OLR: 90 g TS·L^−1^; HRT: 60d	DBP & DMP; 598 mL·L^−1^·d^−1^ 308 mL·L^−1^·d^−1^	Liu et al. (2018)
	Poultry manure		-	Mesophilic conditions at 37 °C; 20-d retention time	285 ml/g VS	Al-Zoubi et al. (2024)
	Kitchen waste + poultry manure		Cow manure	28°C & 37°C; KW:PM ratios of 1:0, 1:1, 2:1, and 3:1	920 mL (mesophilic), 396 mL (RT)	Rahman et al. (2023)
	Chicken manure		Methanogenic archaea, hydrolytic bacteria	Mesophilic conditions; 15% total solids; 5% biochar dosage	314 mL/g VS (biogas)	Ngo et al. (2024)
Biohydrogen	Pine chips	Gasification	Ca_90_Fe_10_/B	700 °C; N₂: 2 L/min; Heating rate: 10 °C/min; Catalyst-to-biomass ratio: 1.5	210.5 mL H_2_/g	Luo and Chen (2024)
	Palm kernel shell		Ni, Ca, K/ modified mordenite (T2-C5)	900 °C; N₂: 100 mL/min; Heating rate: 20 °C/min; Steam/biomass: 2; Water flow: 12.5 mL/h	72.9 mol% H_2_	Acevedo-Paez et al. (2024)
	Pine sawdust	Two-stage fixed-bed steam gasification with in-situ CO_2_ comme	10Ni5Ce/CaO	600 °C; N_2_: 80 mL/h; Heating rate: 20 °C/min; Steam: 5 mL/h; Catalyst ratio Ni/Ce: 2:1	12.8 mmol H_2_/g	Quan et al. (2024)
	Sugarcane bagasse	SCWG	CaO	Gasifier: 1200 °C, 221 bar, SCW/biomass = 0.1; WGS: 150 °C, water/biomass = 0.6; carbonator: 850 °C; CaO/biomass = 1.94; regenerator: 900 °C	14.16% H_2_	Panichkittikul et al. (2024)
	Rice straw	Dark Fermentation	*Clostridium* sp. YM1	RS pretreated with 1% (v/v) HNO_3_ at 121 °C for 15 min; 250 mL Schott bottle, 150 mL working volume; 10% inoculum; 30 °C; initial pH 6.5; 72 h	1.19 mol H_2_/mol sugar	Mahmod et al. ( 2025)
	Cotton-derived sugars		*Shigella flexneri* SPD1	Cotton biomass pretreated with CC:LA NADES (1:9), solid/liquid 1:6, 121 °C for 1 h; enzymatic hydrolysis 48 h at 45 °C; fermentation at 37 °C with 0.5% (w/v) substrate	2.92 mol H_2_/mol hexose (lab scale)2.74 mol H_2_/mol hexose at 4-L working volume	Chandekar et al. (2024)
	Seaweed biomass		*Clostridium* sp. JH03	20 mL serum bottle; 25 °C; pH 9; 72 h	1.71 mol H_2_/mol galactose	Hwang et al. (2024)
	Duckweed biomass		Activated sludge	332 mL; 55 °C; 120 rpm; TS: 5840 mg/L; 168 h	416 mL H_2_/g	Naik and Mohanakrishna (2024)
	DMW-derived Chlorella vulgaris ESP-6 biomass hydrolysate		*Clostridium butyricum* CGS5	1.5% HCl pretreatment, 121 °C, 20 min; 37 °C, 150 rpm, 5% inoculum; pH 5.5	Manual: 211 mL H2/g VS (1.69 mol/mol); Automatic: 179 mL H2/g VS (1.43 mol/mol), max productivity 391.1 mL/L/h	Leong et al. (2024)
	*Eichhornia crassipes*	Anaerobic Fermentation	*Clostridium acetobutylicum *ATCC824	0.5 M NaOH, 121 °C, 60 min; enzymatic hydrolysis at 37 °C for 10 h; fermentation at 37 °C, 10% inoculum, 24 h	2.642 mol H_2_/mol glucose	Aguirre et al. (2024)
Bioelectricity	Wheat bran	Single-chamber MFC	Pantoea dispersa, P. aeruginosa	External resistance: 100 Ω; Internal resistance: 69.5 Ω; Anode: biochar-modified anode (1.5 mg/cm2) ; T°: 37 °C; 2.5 g/L cellulose; 300 mL working volume	9.1 W/m^3^	Kumar et al. (2024)
	Sweet potato waste		Mixed microbial community from domestic wastewater; dominant genera: *Pectobacterium*, *Klebsiella*, *Enterobacter*	External resistance: 500 Ω; Electrode: graphite; pH = 7; 750 g SPW; 1800 mL HQ-wastewater (10 ppm HQ); batch operation, 30 d	1.09 mW/m^2^ max power density; 46.05 mA/m^2^ max current density; 150 mV; 65% HQ removal	Alshammari and Ahmad (2025)
	Corn stalks	MFC–Constructed Wetland (CW)	Mixed electroactive and denitrifying microbial community	70 g/m2 corn stalks; 21.5 L; HRT 48 h; 4.4 mL/min	0.71 mW/m^2^	Ma et al. (2024)
	Sucrose-based synthetic wastewater	Dual-chamber MFC	Anaerobic mixed sludge	COD 3 g/L; 100 mL/chamber; External resistance: 100 Ω; Electrode: carbon cloth; batch; 30 d	150.22 mW/m^2^	Sharma et al. (2021)
	* Synthetic wastewater *		*Scenedesmus obliquus* biocathode + mixed anaerobic sludge anode inoculum	500 mL/chamber*; S. obliquu*s 0.1 g/L; 2300 lx continuous illumination; 15 d; 1000 Ω during continuous operation	13.24 mW/m3 continuous volumetric power density; 1.94 mW/m2 continuous power density; COD removal 92.52%	Bhadra et al. (2024)

Abbreviations: AD, anaerobic digestion; CBP, consolidated bioprocessing; CC, choline chloride; COD, chemical oxygen demand; CSTR, continuous stirred-tank reactor; CW, constructed wetland; DBP, daily biogas production; DMP, daily methane production; DMW, dairy manure wastewater; EM-4, Effective Microorganisms-4; HHF, hybrid hydrolysis and fermentation; HQ, hydroquinone; HRT, hydraulic retention time; LA, lactic acid; MFC, microbial fuel cell; NADES, natural deep eutectic solvent; OLR, organic loading rate; OMW, olive mill waste; RT, room temperature; SBP, sugar beet pulp; SCWG, supercritical water gasification; SHF, separate hydrolysis and fermentation; SPW, sweet potato waste; SSCF, simultaneous saccharification and co-fermentation; SSF, simultaneous saccharification and fermentation; TS, total solids; VS, volatile solids; WGS, water–gas shift.

### Biofuel quality assessment and characterization

Reliable quality monitoring across the biofuel spectrum requires analytical methods tailored to each fuel’s physical state and the operational conditions under which measurements are performed . For liquid biofuels, GC–MS characterizes the FAME composition in biodiesel and detects trace contaminants (residual methanol, glycerol) that can directly affect combustion performance and engine compatibilit.; In lignocellulosic biorefineries, HPLC is used to monitor sugar conversion and ethanol production, as complex carbohydrate profiles strongly influence process efficiency (Bernardes et al. 2021). Benchtop ^1^H NMR coupled to partial least squares regression now predicts oxidative stability of commercial biodiesel in under five minutes without elaborate sample preparation (Conte et al. 2025), a development with direct relevance to cooperative-scale quality control in resource-constrained settings. Biogas quality is quantified by GC for bulk CH_4_ and CO_2_ content, with comprehensive two-dimensional gas chromatography (GC×GC) providing high-resolution trace-compound fingerprinting of complex biogas matrices including sulfur compounds, siloxanes, and halogenated hydrocarbons that determine upgrading requirements (Hilaire et al. 2017).For solid biofuels, thermogravimetric analysis (TGA) quantifies thermal degradation pathways and differential scanning calorimetry (DSC) measures combustion energetics essential for pellet and biochar performance assessment (Shen et al. 2015). The practical challenge in Morocco and across Africa is that most of these instruments assume centralized laboratory access that cooperatives and SME-scale operators do not have. The field-relevant priority is therefore the deployment of portable and low-cost analytical protocols, such as portable FTIR, benchtop NMR, and field-deployable micro-GC validated using locally relevant feedstock matrices, including OMW, olive pomace extracts, and cereal straw hydrolysates. Establishing these protocols would serve a dual function: enabling quality control at the point of production and generating the feedstock characterization datasets that country-specific LCA and techno-economic models currently lack.

### Morocco’s bioenergy transition

Morocco generates approximately 23 million tonnes of crop residues annually, of which less than 2% is currently integrated into modern bioenergy systems (Mahdavi et al. 2023). This gap is not primarily a technology gap, as the conversion pathways described in previous sections are technically available and partially demonstrated on Moroccan feedstocks. It is an institutional, economic, and policy gap, and understanding it requires examining three distinct dimensions: who does the research, what industrial infrastructure already exists, and what structural conditions prevent investment at scale.

#### The academic research base

Moroccan universities have built a substantive bioenergy research base whose contribution is underrepresented in the international literature relative to its actual scope. UM6P in Ben Guerir is the primary hub, with active programs spanning AD systems optimization and biodigester scaling adapted to African conditions (Niya et al. 2024b, 2026), waste to BioH_2_

* via* fermentative and MEC pathways (Ou-douaou et al. 2026), macroalgal biodiversity characterization along Morocco’s Atlantic and Mediterranean coasts as a foundation for marine bioenergy feedstock assessment (El Meraoui et al. 2024; Boutafda et al. 2025), and enzymatic saccharification of regionally relevant lignocellulosic substrates. Mohammed V University in Rabat demonstrated CBP of olive mill waste using indigenous fungi (Nait M’Barek et al. 2020). This is a result with direct commercial relevance to Morocco’s largest agro-industrial waste stream. Institutions in the Fez-Meknes region have characterized forest green waste composition and energy conversion potential (Bendaoud et al. 2022). UM6P maintains active partnerships with French CNRS laboratories and operates OCP-funded research infrastructure representing one of the largest investments in applied chemistry and bioprocess engineering on the African continent. A distinct and geographically differentiated contribution comes from ASARI (UM6P’s African Sustainable Agriculture Research Institute), located at the Laayoune campus in Morocco’s southern regions. ASARI has quantified the AD potential of camel, bovine, goat, and sheep manure across the Guelmim-Oued Noun, Laâyoune-Sakia El Hamra, and Dakhla-Oued Ed-Dahab regions, estimating a theoretical methane potential of approximately 44.9 million m^3^ CH_4_/year, an electrical energy potential of 155 GWh/year, and a recoverable biofertilizer output of approximately 64,400 tonnes of compost per year from digestate (Karouach et al. 2024). The Laâyoune-Sakia El Hamra region alone generates over 312,000 tonnes of livestock manure annually, with camels constituting a significant share of the regional herd. Boubal et al. (2026) further reviewed camel dung valorization via AD and pyrolysis, establishing it as a viable substrate for biogas, bio-oil, and biochar production suited to the circular bioeconomy logic of arid and semi-arid zones. This work positions Morocco’s southern camel-breeding regions as an underexplored bioenergy zone with quantified potential and institutional research capacity already in place. The structural problem is not research output, which is growing across all research institutes, but the near-total absence of industrial partners required to translate that output into commercial bioenergy products. Without companies willing to co-invest in operations at the pilot and demonstration scales, academic findings remain within the research system rather than entering the energy market. This reflects the broader innovation ecosystem deficit identified in the bibliometric and patent analyses: Morocco generates publications but almost no bioenergy patents, and the double-helix linkage between universities and industry that drives technology transfer in mature innovation systems has not yet formed in this sector.

#### Existing industrial platforms

Several Moroccan agro-industrial sectors already valorize biomass residues for energy or material applications. These are not proof-of-concept pilots but rather operational systems embedded in existing supply chains, and they constitute the most realistic foundation for bioenergy scale-up without requiring entirely new infrastructure to be built from scratch. Olive mills across the Meknes-Fez and Marrakech-Safi regions already combust solid pomace for on-site thermal energy, constituting a first-generation bioenergy system with existing logistics, operator familiarity, and established biomass aggregation patterns. The liquid co-product, olive mill wastewater, remains almost entirely unprocessed for energy recovery despite representing the highest-value untapped biogas resource in Moroccan agro-industry. ONEE’s AD of sewage sludge at major urban wastewater treatment plants already produces 21.6 million m^3^ of biogas annually (Fertahi et al. 2023), though grid injection and biomethane upgrading infrastructure remains absent, leaving most of this gas flared or vented. OCP Group has initiated circular bioeconomy linkages through its agricultural subsidiaries, including programmatic interest in digestate-derived biofertilizers that create a natural convergence between Morocco's phosphate economy and emerging AD-based biorefinery models. OCP’s existing distribution networks and farmer relationships provide a ready-made route to market for biofertilizer co-products that standalone biogas operators would need years to establish independently. The Mediouna landfill biogas capture facility near Casablanca, operated under a public-private arrangement through the National Waste Management Programme (NWMP), is Morocco’s most technically advanced private sector bioenergy operation and demonstrates that the regulatory and financing structures for biogas-to-electricity can function in the Moroccan context when the feedstock is sufficiently large and centralized. Camel herding and mixed livestock production dominate the agricultural economy of the Laâyoune-Sakia El Hamra, Guelmim-Oued Noun, and Dakhla-Oued Ed-Dahab regions, generating a combined manure stream quantified by ASARI at over 580,000 tonnes/year across the three zones (Karouach et al. 2024). Unlike OMW, camel and mixed livestock manure is a dispersed solid substrate better suited to decentralized biodigester models at pastoral community or cooperative scale, precisely the deployment model that has shown the most resilience in East African biogas programs. The biofertilizer co-product stream is estimated at 64,400 tonnes/year of compost equivalent from digestate. This is particularly relevant in the context of southern Morocco’s arid soils, where organic matter amendment is both scarce and strategically valuable for supporting forage crop production and combating land degradation.

#### Structural barriers

The structural conditions blocking Morocco’s bioenergy transition from potential to deployment are well defined, and several have now been confirmed in the policy literature as explicit regulatory gaps rather than merely observed absences. Morocco’s 2021 National Biomass Energy Strategy, announced by the Ministry of Energy with an allocated budget of 4.3 billion Moroccan dirhams (approximately 430 million euros), acknowledged the country’s gross energy potential from biomass at 13 TWh/year. This includes 6 TWh from the agricultural sector alone, compared with the current exploitation of less than 1% of this potential. The strategy is structured around three sequential pillars: establishing the regulatory and legislative framework for bioenergy recovery (2021–2024); deploying pilot projects and building technical capacity (2024–2028); and implementing financing mechanisms and incentive measures for full commercial-scale development (2024–2030). Under its most ambitious scenario, Morocco targets 28 TWh of biomass-derived electricity by 2050, representing 13% of total projected electricity generation (Mana et al. 2021). The existence of this strategy demonstrates that the policy objectives are formally established; however, their effective implementation remains limited. t the investment level, a major barrier is the absence of a biogas-specific feed-in tariff or equivalent fiscal instrument. Morocco's renewable energy framework (Law 13.09) established the legal basis for renewable electricity production and self-consumption, and the Moroccan Agency for Sustainable Energy (MASEN) and ANRE institutional architecture has successfully attracted large-scale private solar and wind investment. However, biogas to grid injection remains without a dedicated price support mechanism, meaning that small and medium operators cannot secure project financing on the basis of a guaranteed offtake price. This is not a technical constraint but a regulatory one that requires coordination between the Ministry of Energy, ANRE, and ONEE to resolve. Without a feed-in tariff structured to reflect avoided methane emissions, tax incentives , waste management cost savings, and the rural energy access premium, bioenergy projects remain commercially marginal relative to fossildespite Morocco’s 90–95% hydrocarbon import dependence (Lebrouhi et al. 2024). The structural fragmentation of the olive milling sector compounds this problem. Individual mill capacity in Morocco is typically too small to justify standalone biogas investment. For instance, the OMW volume per mill falls below the threshold that renders a UASB reactor economically viable on a single-operator basis. The economics only become favorable at cluster scale, where OMW streams from multiple mills are aggregated and co-treated in shared infrastructure. This cooperative cluster model has already proven viable in comparable Mediterranean olive-belt contexts. Southern Spain’s Andalusian biogas program and Tunisia’s cooperative OMW treatment initiatives demonstrate that it can work when the regulatory reclassification of OMW from waste to resource removes the disposal cost obligation and creates a positive economic incentive (Comparetti et al. 2025). Replicating this in Morocco requires both the regulatory reclassification and a cooperative financing vehicle aligned with smallholder ownership structures rather than single-operator project finance norms. A further barrier is the absence of localized feedstock data for biomass streams outside the olive and citrus belts. This data gap concerns cereal straw, date palm residues, and livestock waste across Moroccan regions, including the quantitatively significant camel-manure resource identified in southern Morocco by Karouach et al. (2024). The spatial distribution, seasonal availability patterns, moisture content variability, and competing uses have not been systematically characterized at the resolution needed to underwrite biodigester investment. Without this baseline, both project developers and investors cannot size systems, model cash flows, or assess resource security over a financing horizon. The ASARI livestock manure quantification for the southern regions represents a partial answer to this data gap, but equivalent spatial mapping is needed for cereal-producing regions of the north and center and for the date palm cultivation zones of the southeast.

## Life cycle assessment of bioenergy technologies

A thorough sustainability assessment of bioenergy systems requires the application of LCA to evaluate environmental impacts such as energy balance, GHG emissions, and resource use. LCA serves as a critical decision-support tool for policymakers and stakeholders, helping guide system optimization and inform sustainable investment strategies (Osman et al. 2024). Global LCA studies on biofuels, biogas, and BioH_2_ have revealed diverse environmental outcomes depending on the feedstock, production process, and regional context (Sahota et al. 2024). For example, bioethanol production from banana waste has a greater environmental impact than production from potato and papaya residues. However, process optimization, renewable energy use, sustainable pH adjustment, and enzyme reuse can substantially improve its environmental sustainability (Fagundes el al. 2024). Innovative approaches, such as co-production strategies, can reduce environmental burdens by producing multiple outputs from a single system. However, LCA studies on bioenergy in Africa remain limited. While countries like Morocco have initiated assessments of hybrid energy systems (*e.g*., solar and fuel cells), others, such as Nigeria, have seen minimal LCA research on bioenergy (Isah et al. 2025). One life-cycle study of integrated bioethanol production combined with herbal mushroom (*Sanghuangporus vaninii*) cultivation from regional biomass found that the co-production system reduced GHG emissions by 148.68% relative to a single-output bioethanol process (Therasme et al. 2021). That is, the mushroom co-product credit rendered the integrated system net-negative in global-warming potential.

BioH_2_ production is presently gaining attention due to its high energy efficiency and potential to reduce GHG emissions. However, challenges remain, particularly regarding high production costs and energy-intensive methods. Many LCA studies emphasize the need for technological advancements to improve the scalability and economic viability of BioH_2_, particularly in low-income regions (Thirumalaivasan et al. 2024). Furthermore, integrating bioenergy production with circular bioeconomy practices, such as waste valorization and nutrient recycling, can generate additional environmental and economic benefits. The environmental impact of bioenergy systems is highly dependent on local conditions, including feedstock availability, technological maturity, and the management of by-products. Consequently, region-specific LCA studies are essential to fully capture the sustainability potential of bioenergy within circular bioeconomy frameworks.

## Circular bioeconomy concepts in bioenergy systems

The circular bioeconomy provides a framework for producing bioenergy in a more sustainable and resource-efficient manner by emphasizing waste valorization and closed-loop systems (Shaba et al. 2024). This approach is highly relevant in the global south, where energy demand is rising while waste management systems remain underdeveloped (Ogwu et al. 2025). In many of these regions, waste is still largely disposed of through open dumping, in contrast to the global north, where more advanced technologies enable material recovery and recycling (Anshassi and Townsend 2025). Integrating bioenergy into circular bioeconomy strategies can therefore help optimize resource use while reducing environmental impacts. . Integrating biomass recovery with waste minimization approaches within circular systems of reuse and recycling makes it possible to efficiently transform waste into useful resources for bioenergy production. This synergy helps to improve resource efficiency, reduce material losses and enhance the sustainability of energy systems. The combination of these two approaches is therefore a key driver for the development of sustainable and efficient bioenergy systems (Anacleto et al. 2025). In practice, this approach seeks to minimize waste while maximizing material reuse within production cycles. Biomass residues can be redirected toward biofuel and biogas production, while by-products such as digestate and CO_2_ can be repurposed for agricultural and industrial applications, creating more efficient and sustainable resource loops (Kumba et al. 2024). Achieving this transition in Africa requires sustained investment, supportive policy frameworks, and international collaboration (Leela et al. 2024). In Morocco, circular bioeconomy efforts are gaining momentum, with advancements in recycling, resource efficiency, and sustainable practices across key sectors, including agriculture and waste management (Diaco et al. 2020). However, the integration of bioenergy into this framework remains limited. Although circular bioeconomy initiatives are emerging across Africa, most efforts have concentrated on waste management, sustainable agriculture, and rural development, with relatively limited application to bioenergy production (Leela et al. 2024). Several case studies highlight the progress achieved so far. For example, EcoPost in Kenya represents a successful waste-to-resource initiative that converts post-consumer plastic waste into durable construction materials, effectively reducing pollution and generating green jobs (Mbamalu and Mohammed 2025). In Ethiopia, Oxfam’s 2014 initiative integrates sustainable agricultural practices with waste minimization and gender empowerment by training smallholder farmers to repurpose agricultural waste as fertilizer and livestock feed, thereby enhancing productivity and resilience to climate change. Similarly, Gudie Leisure Farm in Uganda adopts an agroforestry model that combines food and cash crop cultivation with tree planting and organic practices to promote environmental sustainability and local livelihood development. While these initiatives illustrate growing momentum in circular bioeconomy implementation across diverse sectors, they largely overlook the bioenergy component, which highlights a critical gap and the opportunity to develop integrated bioconversion systems that transform biomass into renewable energy sources. Adopting circular bioeconomy principles in bioenergy systems delivers significant socioeconomic and environmental benefits. On the socio-economic side, bioenergy projects generate employment across the entire value chain, from feedstock collection and processing to energy distribution (Leela et al. 2024). For example, India installed biodigesters with an estimated biogas production capacity of 3.635 billion m^3^/year, contributing torural empowerment and enhancing livelihoods (Singh and Kalamdhad 2023). Similarly, in Kenya a nd Tanzania, converting agricultural residues into bioenergy not only reduces the amount of waste but also provides farmers with supplementary income, improving financial stability and contributing to rural development (Hamid and Blanchard 2018; Baitu et al. 2024). Improved energy access is another socio-economic advantage. In Ethiopia, decentralized biogas digesters have provided rural households with reliable sources of energy for cooking and lighting, reducing dependence on firewood and enhancing living conditions (Wassie and Adaramola 2020). In South Africa, agricultural residues have been utilized to improve energy security and create new economic opportunities, especially in rural areas (Batidzirai et al. 2016). From an environmental perspective, bioenergy systems mitigateclimate change through significant reduction in GHG emissions. For instance, Afotey and Sarpong (2023) estimated that slaughterhouses waste in Ghana has the potential to produce 6.792 × 10^9^ m^3^ of biogas annually, equivalent to 7.110 × 10^9^ kWh of electricity, or 43.01% of the country’s annual electricity consumption. This could prevent the release of 1.075 × 10^10^ kg of CO_2_ and yield 4.514 × 10^8^ kg of biofertilizer, promoting sustainable agriculture.

## Policy recommendations for advancing biomass and bioenergy in Africa

According to the Africa Bioenergy Policy Framework and Guidelines (UNECA 2013)**,** the successful implementation of biomass and bioenergy initiatives in Africa depends on well-structured policies thatalign with climate goals, address energy demands, and take into account the diversity of biomass resources across countries and regions. The current lack of policy consistency and regulatory alignment among nations hinders the development of a unified bioeconomy (Ogwu et al. 2025).

### Policy alignment with climate change goals, energy needs, and biomass availability

Effective biomass energy policies must be aligned with both national and international climate commitments, including the Paris Agreement and the Sustainable Development Goals. Such alignment is essential to support the energy transition and strengthen energy security, particularly in regions with abundant biomass resources. Developing national biomass inventories can help governments identify available feedstocks, assess their sustainable utilization potential, and reduce potential conflicts with food production (Tihamer Tibor and Benedek 2025). Bioenergy strategies should be tailored to the specific resource endowments, energy needs, and socioeconomic conditions of each country. For instance, in countrie such as Nigeria and the Democratic Republic of Congo, where electricity shortages are a pressing concern, policies should prioritize bioelectricity generation from agricultural residues and municipal waste. Countries that rely heavily on imported fossil fuels, including Morocco, Kenya, and Senegal, could promote the conversion of suitable organic waste streams into biofuels and other forms of bioenergy. Nations with strong agricultural sectors, such as Ethiopia and Nigeria, can integrate biomass energy into sustainable farming by converting crop residues and livestock waste into biogas and biofertilizers. Rapidly urbanizing countries, such as South Africa and Egypt, may benefit from waste-to-energy solutions that simultaneously address municipal waste-management challenges while contributing to local energy supply. Furthermore, economic instruments like carbon pricing and emissions trading can incentivize cleaner bioenergy solutions and attract investment in advanced biomass technologies (Su’ait et al. 2025).

### Integrated biorefineries and circular bioeconomy

Promoting bioenergy within a circular bioeconomy framework requires integrated waste management policies that prioritize poly-generation systems. Such systems convert biomass residues into multiple high-value products. For example, digestate can serve as a nutrient-rich fertilizer (Chen et al. 2024), vinasse can serve as a feedstock for biogas or bioplastics (Madhuri et al. 2025), and pretreatment residues can be converted into biochar for carbon sequestration and soil enhancement (Dessie et al. 2025). Realizing this potential requires investment in advanced processing technologies, supportive infrastructure, and regulatory frameworks that promote the circular use of biomass. Integrated waste management must also address the full biomass lifecycle, from feedstock cultivation through energy production to waste disposal and resource recovery, ensuring that residues are treated as valuable resources rather than waste. Government incentives, such as subsidies for resource recovery technologies, funding for research and innovation, tax breaks for waste-to-value enterprises, and regulations mandating recycling or upcycling of bioenergy residues, can accelerate the transition toward a circular bioeconomy (Ogwu et al. 2025).

### Integration with existing energy infrastructure

The adoption of bioenergy technologies in Africa depends heavily on their integration into existing power grids, transportation networks, and industrial energy systems. Many countries face challenges due to outdated or underdeveloped infrastructure, which limits large-scale bioenergy deployment (Leal Filho et al. 2024). Policy priorities should therefore include grid modernization and expansion to accommodate bioelectricity generated from biomass. Most existing studies focus on individual biofuels or isolated technological pathways, often without a systems-level perspective. Table 2 presents a comparison of reviews covering bioenergy in African contexts.

**Table 2:** Comparative features of bioenergy review studies in Africa and Morocco

Type of biomass	Bioenergy type	Conversion technology	Bibliometric analysis	Circular economy	LCA	Integrated biorefinery	Morocco focus	Biomass mapping	References
Agricultural residues	Bioenergy	Waste-to-Energy	×	✔	×	×	×	✔	Uzoagba et al. (2024)
Olive Mill Wastewater (OMWW)	Biogas, Biofertilizer	Recycling & Valorization	×	✔	×	✔	✔	×	Haouas et al. (2022)
Algal biomass	Biodiesel, Bio-oil, Biogas	Algal Bioconversion	×	×	×	×	✔	×	Wahby et al. (2018)
Agro-wastes, Xenobiotics, dyes	Bioelectricity	Microbial Fuel Cells (MFCs)	×	×	×	✔	×	×	Chaturvedi and Verma (2016)
Animal manure	Biogas	Anaerobic Digestion	×	×	×	×	×	×	Sibanda and Uzabakiriho (2024)
Solid waste (mixed)	Bioenergy	Waste-to-Energy	×	✔	×	✔	×	×	Kumba et al. (2024)
Lignocellulosic biomass	Bioethanol	Fermentation	×	×	×	×	×	✔	Gares et al. (2020)
Multiple (Agricultural residue, Organic waste, Livestock, Algae etc.)	Bioethanol, Biogas, Biohydrogen , Bioelectricity	Bioconversion	✔	✔	✔	✔	✔	✔	This review

## Conclusion and future outlook

This review examined why Africa's substantial biomass resources have not translated into modern bioenergy capacity. The synthesis of technological, bibliometric, patent, and life-cycle evidence indicates that the principal barriers are institutional and economic rather than technical. Although the conversion processes underlying bioethanol, biodiesel, AD, BioH_2_, and MFCs have been demonstrated and, in several cases, validated using African feedstocks, scaling these technologies beyond the laboratory remains challenging. Agricultural residues exhibit substantial variability in composition and properties, while comprehensive feedstock characterization, standardized pretreatment protocols, and region-specific LCA and TEA data remain limited. The contrast between bibliometric growth and patent activity reinforces this diagnosis: African research output is increasing rapidly, whereas patenting remains negligible, revealing a persistent disconnect between a maturing academic base and limited industrial translation. Among these pathways, AD represents the most promising near-term option for African bioenergy development, particularly for wet agricultural residues, because it produces nutrient-rich digestate as a valuable agricultural co-product. In Morocco, its integration with the country's phosphate-based agricultural economy creates additional opportunities for valorizing waste streams such as OMW and camel manure. However, wider deployment will depend primarily on supportive policies, appropriate economic incentives, and reliable, spatially resolved feedstock inventories rather than on further technological development alone. The principal contribution of this review is therefore not to provide a catalogue of conversion technologies, but to reframe Africa's bioenergy challenge. Closing the continent's bioenergy gap will require innovation-ecosystem linkages, price-support instruments, and country-specific sustainability data capable of converting demonstrated scientific results into financeable projects. Future research should prioritize spatially resolved feedstock mapping, region-specific LCA and TEA, standardized feedstock characterization and pretreatment protocols, and cooperative financing models. These enabling measures should proceed alongside frontier research on nanomaterial-enhanced microbial systems and advanced pretreatment, thereby allowing circular bioeconomy integration to function as an operational design principle.

## Data Availability

All data generated or analyzed during this study are included in this published article
